# Activating CD137 Signaling Promotes Sprouting Angiogenesis via Increased VEGFA Secretion and the VEGFR2/Akt/eNOS Pathway

**DOI:** 10.1155/2020/1649453

**Published:** 2020-10-24

**Authors:** Bo Li, Yue Zhang, Runting Yin, Wei Zhong, Rui Chen, Jinchuan Yan

**Affiliations:** Department of Cardiology, Affiliated Hospital of Jiangsu University, Zhenjiang, China

## Abstract

Combination of antiangiogenesis and immunotherapy may be an effective strategy for treatment of solid tumors. Our previous work reported that activation of CD137 signaling promotes intraplaque angiogenesis. A number of studies have demonstrated that vascular endothelial growth factor receptor 2 (VEGFR2) is a key target for angiogenesis. However, it is unknown whether CD137-mediated angiogenesis is related to VEGFR2. In this study, we investigated the effect of CD137 on the VEGFR2 expression and explored the underlying mechanisms of CD137-mediated angiogenesis. Knock-out of CD137 in ApoE^−/−^ mice significantly decreased neovessel density in atherosclerotic plaques. CD137 silencing or inhibition attenuated endothelial cell (ECs) proliferation, migration, and tube formation. We found activation of CD137 signaling for increased VEGFR2 transcription and translation steadily. Moreover, CD137 signaling activated phosphorylated VEGFR2 (Tyr1175) and the downstream Akt/eNOS pathway, whereas neutralizing CD137 signaling weakened the activation of VEGFR2 and the downstream Akt/eNOS pathway. The aortic ring assay further demonstrated that CD137 signaling promoted ECc sprouting. Inhibition of VEGFR2 by siRNA or XL184 (cabozantinib) and inhibition of downstream signaling by LY294002 (inhibits AKT activation) and L-NAME (eNOS inhibitor) remarkably abolished proangiogenic effects of CD137 signaling both in vitro and ex vivo. In addition, the condition medium from CD137-activated ECs and vascular endothelial growth factor A (VEGFA) had similar effects on ECs that expressed high VEGFR2. Additionally, activating CD137 signaling promoted endothelial secretion of VEGFA, while blocking CD137 signaling attenuated VEGFA secretion. In conclusion, activation of CD137 signaling promoted sprouting angiogenesis by increased VEGFA secretion and the VEGFR2/Akt/eNOS pathway. These findings provide a basis for stabilizing intraplaque angiogenesis through VEGFR2 intervatioin, as well as cancer treatment via combination of CD137 agonists and specific VEGFR2 inhibitors.

## 1. Introduction

Angiogenesis is an intricate process involving basement membrane degradation, as well as endothelial cell activation, proliferation, and migration to form new vessels [1]. Neovessels are essential for providing sufficient amount of nutrients and oxygen for proliferating cells in hyperplastic diseases [2, 3]. As the main cause of atherosclerotic plaque hemorrhage and rupture, angiogenesis promotes adverse cardiovascular events, such as myocardial infarction, which is a serious issue during treatment [4]. Although many angiogenesis inhibitors have been developed, the process of pathological angiogenesis is quite complex and involves multiple regulatory factors. Therefore, further research is needed to identify other potential therapeutic targets for angiogenesis-related diseases.

CD137 (also known as ILA/4-1BB/TNFRSF9) is an important immune checkpoint molecule, which is expressed on immune cells, myeloid cells, and vascular cells, such as ECs in an activation-dependent manner. In addition, CD137 is a well-known T cell costimulatory molecule [5, 6]. There is accumulating evidence for the expression of CD137 in human atherosclerotic plaque lesions [7]. The CD137 expression in inflamed tissues was found to be induced in mural cells, such as vascular ECs and smooth muscle cells, after stimulation with proinflammatory cytokines [7–9]. The ligand of the CD137 receptor, CD137L (4-1BBL), is mainly expressed by antigen-presenting cells, such as dendritic cell macrophages and activated T cells [3]. CD137L binding with CD137 on ECs could trigger a cascade of inflammation and immune responses in atherosclerotic plaque [7, 10, 11]. We previously reported that activation of CD137 signaling promoted angiogenesis in the plaque of ApoE^−/−^ mice through different pathways [12, 13]. However, it remains unclear whether CD137 plays a key role in angiogenesis.

The expression level of CD137 in tumor vessels highly correlates with the tumor's degree of malignancy [14]. Therefore, the strategy for cancer should include a combination of antiangiogenesis and immunotherapy [15, 16]. The experience of application of CD137 agonists and angiogenesis inhibitors in tumor therapy [17–22] may suggest that inhibition of angiogenesis might be useful also for CD137-expressing stable atherosclerotic plaque. Thus, it is becoming increasingly important to further explore how CD137 signaling regulates angiogenesis in atherosclerosis.

Angiogenesis encompasses several stages, including basement membrane degradation, ECs migration, and proliferation, as well as recruitment of mural cells. Migrating tip cells along with proliferating stalk cells form a vascular sprout, and endothelial tip cells of two sprouts converge to form a neovessel [23, 24]. The process of sprouting involves sensing and guiding roles of endothelial tip cells with long, dynamic filopodia. Specifically, VEGFR2, mainly expressed in filopodia, can sense environmental stimulators, such as VEGFA. Migrating tip cells are followed closely by proliferative stalk cells that extend the sprouts together [23]. Zarkada et al. reported that VEGFR2 is indispensable for postnatal angiogenesis, and even a little VEGFR2 is able to sustain angiogenesis to some extent [25].

VEGFR2-induced angiogenesis involves intricate signaling pathways [26–28]. Activated VEGFR2 indicated by the phosphorylated 1173 site in mice (corresponding to 1175-Tyr in humans) is crucial for endothelial and hematopoietic cell development [29]. VEGFA, which is secreted by tumor cells and ECs, is a potent inducer of sprouting angiogenesis [26, 27, 30, 31] through VEGFR2 [32]. By upregulating the expression of VEGFA [33], inflammation contributes to initiation of angiogenic process. Although VEGFR2 seems critical in atherosclerosis, its effects are still controversial [32]. Moreover, it remains unknown whether VEGFA and VEGFR2 signaling is implicated in CD137 signaling-mediated angiogenesis.

Here, we detected angiogenesis-related factors and found that VEGFR2 increased steadily in both transcription and translation levels after activation of CD137 signaling. These findings prompted us to explore the underlying mechanisms of CD137 regulation of the VEGFR2 expression.

## 2. Materials and Methods

### 2.1. Ethics Statement and Animals

Animal experiments conducted in this study were reviewed and approved by the Animal Care and Use Committee of Jiangsu University. Six- to eight-week-old, male, wide-type C57BL/6 J mice, weight 20-22 g, were purchased from the Animal Center of Jiangsu University. Six- to eight-week-old, male, 20-22 g, ApoE^−/−^ and ApoE^−/−^CD137^−/−^ mice were obtained from the Nanjing Biomedical Research Institute of Nanjing University (Nanjing, China). C57BL/6 J mice were fed with a normal diet, and ApoE^−/−^ mice were fed with a high fat diet. All of the mice were provided with water ad libitum.

### 2.2. Cell Culture

Human umbilical vein endothelial cells (HUVECs) applied in the tube formation assay were maintained in ECM (Endothelial Cell Medium, ScienCell) supplemented with 10% FBS, 100 U/mL 100 U/mL penicillin, and 100 mg/mL. Mouse brain microvascular endothelial cells (MBVECs) were cultured in Dulbecco's Modified Eagles Medium (DMEM) (Sigma-Aldrich) supplemented with 10%FBS (Sigma-Aldrich), 100 U/mL penicillin, and 100 mg/mL streptomycin (Sigma-Aldrich). Cells were maintained in an incubator at 37°C in humidified atmosphere with 5% CO_2_ and subjected to serum starvation overnight before different treatments.

### 2.3. Mouse Model and Cell Treatments

ApoE^−/−^ and ApoE^−/−^CD137^−/−^ mice were fed with high fat diet until the 19th week. The following reagents were used in this study: TNF*α* (10 ng/mL, PeproTech), recombinant murine VEGF165 (20 ng/mL, PeproTech), recombinant CD137L (10 *μ*g/mL, Sangon Biotech), human 4-1BB/TNFRSF9/CD137 antibody (10 *μ*g/mL, R&D), XL184 (cabozantinib, an inhibitor of VEGFR2 which was applied to the tube formation assay and aortic ring assay, 0.03 *μ*M, MedChemExpressan), LY294002 (an inhibitor of p-AKT, 5 *μ*M, MedChemExpress), siRNA-CD137 (100 nM, GenePharma), siRNA-VEGFR2 (50 nM, GenePharma), CD137 (4-1BB) Monoclonal Antibody (10 *μ*g/mL, eBioscience), and L-NG-nitroarginine Methyl Ester (L-NAME, eNOS inhibitor, 100 *μ*M, MedChem Express).

### 2.4. Small Interfering RNA Transfection

For CD137 and VEGFR2 silencing, murine CD137-specific small interfering RNA (siRNA, 100nM, GenePharma) (sense, 5′-GCU GCC CUC CAA GUA CCU UTT-3′; antisense, 5′-AAG GUA CUU GGA GGG CAG CTT-3′), VEGFR2 siRNA (50 nM, GenePharma) (5′-GAG CAU GGA AGA GGA UUC UTT-3′; 5 ′-AGA AUC CUC UUC CAU GCU CTT-3′), and negative control (sense, 5′-UUC UCC GAA CGU UGC ACG UTT-3′; antisense, 5′-ACG UGA CAC GUU CGG AGA ATT-3′) were transfected into murine vascular endothelial cells with Lipofectamine™ 3000 Transfection Reagent (40 pmol, Invitrogen) for 48 hours to detect protein levels by western blot. Negative control FAM (carboxy-fluorescein) was applied to assess the transfection efficiency under the fluorescence microscope.

### 2.5. Immunohistochemistry Staining

At the age of 19 weeks, ApoE^−/−^ and ApoE^−/−^CD137^−/−^ mice were euthanized by cervical dislocation, and thoracic aorta was removed. Serial sections on the same paraffin blocks were applied to perform immunohistochemistry staining by SP Rabbit & Mouse HRP Kit (DAB) (CWbiotech). The slides were boiled for 10 minutes with EDTA (ethylene diamine tetraacetic acid) antigen retrieval buffer, blocked with normal goat serum, and incubated with primary antibody for CD31 (1 : 100, Abcam) at 4°C overnight. The following day, the slides were treated with biotin-linked goat anti-rabbit/mouse IgG, followed by Streptavidin-HRP. DAB chromogen solution was used to develop the color (positive cells stained brown), while hematoxylin was applied to counterstain cell nuclei. Brown areas were calculated using Image-Pro Plus 8.0 (Media Cybernetics).

### 2.6. Mouse Aortic Ring Assay

Aortic rings of C57BL/6 J mice were obtained in line with the protocol [34]. Briefly, after euthanasia, thoracic aorta was exposed and removed. We peeled the aortic adventitia and cut the rest of aorta into 1 mm wide rings. The rings were treated with different treatments according to the grouping after overnight serum starvation and transferred into a 96-well plate which preembedded with type I collagen (1 mg/mL, Millipore) containing rmCD137L, p-Akt inhibitor (LY294002), p-eNOS inhibitor (L-NAME), or/and VEGFR2 inhibitor (XL184, cabozantinib). About 2.5% fetal bovine serum (Gibco) replaced fresh medium every other day until the seventh day. The sprouts were photographed by a microscope (Olympus), and the number of sprouts was analyzed using Image-Pro Plus 8.0.

### 2.7. Immunofluorescence of Aortic Rings

On day 7 after embedding aortic rings with corresponding treatments in rat tail collagen type I, we removed the upper culture supernatant and washed with a solution containing phosphate buffer saline (PBS), CaCl_2_, and MgCl_2_. The following step involved fixation with 100 *μ*L of 4% paraformaldehyde (PFA) per well at room temperature (RT) for 1 hour, followed by permeabilization with the solution containing PBS, CaCl_2_, MgCl_2_, and 0.25% Triton X-100, at RT for 30 minutes twice. Blocking with 100 *μ*L of 10% bovine serum albumin (BSA) was performed at 37°C for 1 hour. Subsequently, diluting BS1 lectin-FITC (0.1 mg/mL) in PBLEC (PBS, CaCl_2_, MgCl_2_, 0.1 ml of 1 M MnCl_2_ solution and 1% Tween-20) was performed to stain ECs. They were incubated with 50 *μ*L per well at 4°C overnight. The whole plate was washed three times in PBS+0.1% Triton X-100 at RT for 30 minutes. 4′,6-diamidino-2-phenylindole (DAPI, 100 ng/mL) was used to stain cell nuclei. Finally, we washed the aortic rings with distilled water once and photographed them under the fluorescence microscope.

### 2.8. Endothelial Cell Tube Formation

HUVECs of 3-6 passages were applied in the tube formation assay [35]. Specific treatments were applied to HUVECs as for the aortic rings. When HUVECs reached 80% confluence, about 70 *μ*L/well of growth factor-reduced (GFR) Matrigel basement membrane matrix (BME, Corning) was added into a precooled 96-well plate at 37°C for 30 minutes. In the meantime, HUVECs were digested with 0.25% trypsin containing EDTA. HUVEC pellets were resuspended at a concentration of 3 × 10^5^ cells per mL and added 100 *μ*L/well into BME embedded 96-well plate, then kept immobile for 1 h in a humid incubator with 37°C and 5% CO_2_. We checked the plate every hour until the proper tube formation was observed. We captures images with inverted microscopy and quantified the total tube length and branches using Image-Pro Plus 8.0 software.

### 2.9. Total RNA Isolation and Real-Time PCR (RT-PCR)

Endothelial total RNA was extracted with the TRIzol reagent (Invitrogen, USA). HiScript® Q Reverse Transcriptase SuperMix (Vazyme, China) was applied for cDNA synthesis and then amplified 1 *μ*g total RNA with the AceQ® qPCR SYBR® Green Master Mix (Vazyme, China) by the Roche LightCycler 480 (Roche, Germany). The relative mRNA level of each gene was standardized by *β*-actin. The primer pairs (Sangon Biotech, China) used in this experiment are listed in the supplementary materials.

### 2.10. Western Blotting

ECs were plated and harvested after undergoing the required treatments. Total cell lysate was extracted with radioimmunoprecipitation assay buffer (RIPA) and protease inhibitor cocktail (CWbiotech) and then quantified by the BCA Protein Assay Kit (CWbiotech). After heating at 95°C mixed with 5× loading buffer for 8 minutes, the equivalent protein was loaded into SDS-PAGE gel (EpiZyme) at 70 mV, followed by 110 mV. Subsequently, we transferred protein of gel to polyvinylidene fluoride (PVDF, 0.22 *μ*m pore size, Millipore) membranes at 350 mA for 2 hours. We blocked PVDF membrane with 5% nonfat milk or 5% BSA in 1× TBST (tris-buffered saline containing 0.1% Tween-20) at 37°C for 1 hour. Further step included incubation with the following primary antibodies at 4°C overnight: CD137 (1 : 1000, Abcam), VEGFR2 (1 : 1000), phospho-VEGF receptor 2 (Tyr1175/1173) (1 : 1000, CST), Akt (1 : 1000, CST), phospho-Akt (Ser473) (1 : 1000, CST), eNOS (1 : 1000, CST), phospho-eNOS (Ser1177) (1 : 1000, Abcam), VEGFA (1 : 500, Proteintech), and *β*-actin (1 : 2000, CST). After incubation with Rabbit/Mouse HRP-linked secondary antibodies (1 : 5000, CST), enhanced chemiluminescence (ECL, Millipore) was applied to show blots with the Amersham Imager 600 (GE) machine. The gray value of blots was quantified using ImageJ software.

### 2.11. EdU Cell Proliferation Assay

We added equal volume 2× 5-ethynyl-2′-deoxyuridine (EdU) into EC medium and cultured for another 2 hours. After removing the supernatant and fixing with 4% PFA at RT for 30 minutes, we washed three times with 1× PBS for 15 minutes. Following permeabilization with 0.3% Triton X-100 for 30 minutes at RT, we washed twice with 1 × PBS. We prepared the Click solution in accordance to the instruction of the BeyoClick™ EdU Cell Proliferation Kit with Alexa Fluor 555 (Beyotime, China), incubated for 30 minutes in dark, and then counterstained with 1× Hochest 33342 solution for 10 minutes in dark RT. A fluorescence microscope was used for observation and figure capturing.

### 2.12. Transwell Migration Assay

ECs were cultured in 24-well transwell plates (pore size: 8 *μ*m, Corning) for 12 hours after the treatments. They were fixed with 4% PFA for 30 minutes at RT, washed twice with 1× PBS, stained with 0.1% crystal violet for 1 hour, wiped off the remaining crystal violet of the upper polyethylene terephthalate (PET) membrane, and washed three times before leaving to dry naturally.

### 2.13. Enzyme-Linked Immunosorbent Assay (ELISA)

The VEGFA level in the supernatant was measured with an ELISA kit (MULTI SCIENCES, China) in accordance with the manufacturer's instructions. Briefly, we used centrifuge at 300 g for 10 minutes to remove precipitate. The procedure included use of serial dilution standard, detection antibody, conjugated streptavidin-HRP, and TMB liquid substrate. Incubation was done at RT for 20 minutes. We added 100 *μ*L 1 M HCl stop solution to each well. We monitored the color development with an ELISA plate reader at 450 nm with wavelength correction set at 620 nm.

### 2.14. Statistical Analysis

Each experiment was performed at least three times. All of the analyses were performed using SPSS 23 software. The data were reported as mean ± standard deviation (SD). To compare between control and treated groups, we used two-tailed Student's *t*-test or one-way ANOVA (LSD *t*-test). *P* values <0.05 were considered to be statistically significant.

## 3. Results

### 3.1. Endothelial CD137 Is Critical to Intraplaque Angiogenesis

**Figure 1:**
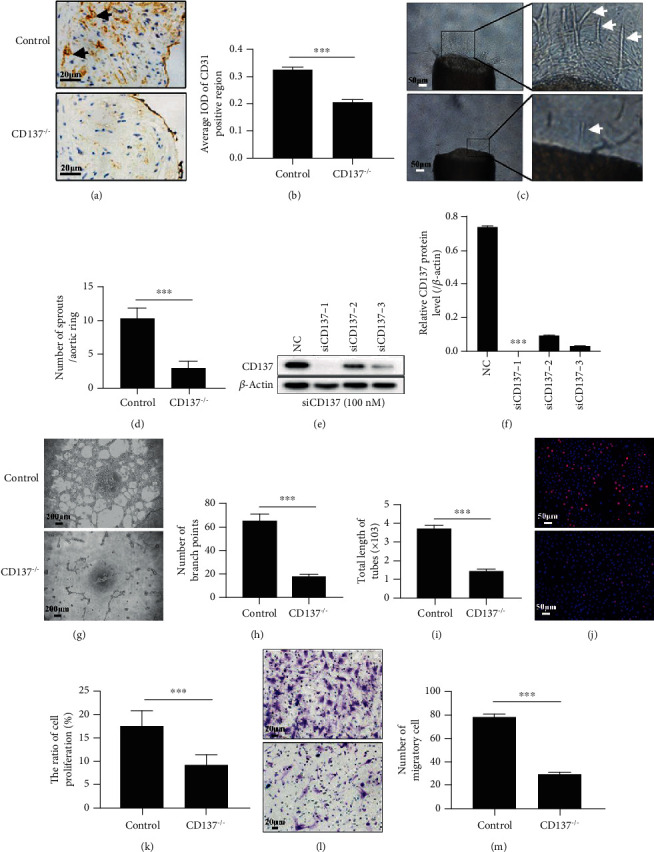
Knocking out/down endothelial CD137 impaired intraplaque angiogenesis. (a) The CD31 expression in ApoE^−/−^ CD137^−/−^ mice and ApoE^−/−^ mice detected by immunohistochemistry in aortic paraffin sections is shown in brown color (black arrows indicate positive cells). (b) CD137 knock-out decreased CD31-positive areas significantly compared with the control. (c) Aortic rings sprouting in CD137^−/−^ and C57BL/6 J mice (*n* = 5 for each group). (d) CD137 knock-out decreased the sprout number compared with the control aortic ring. (e) The silencing efficiency of CD137 siRNA-1, -2, and -3 (100 nM) detected by western blot. (f) siCD137-1 obviously silenced the expression of the CD137 protein. (g) Endothelial cell tube formation in siCD137 and control HUVECs. (h, i) The number of branch points and total length of tubes were both markedly decreased in CD137-silenced human umbilical vein endothelial cells (HUVECs). (j, l) The EdU-555 proliferation assay and transwell assay were applied to detect the proliferative and migratory ECs. (j) Red and blue fluorescence represents proliferating cells and nuclei, respectively. (l) The violet color represents migrating cells stained with 0.1% crystal violet. (k, m) The numbers of proliferative and migratory ECs extremely decreased in CD137-silenced ECs. After siRNA transfection, ECs were treated with TNF*α* for 24 h then applied to western blot, proliferation, and migration assay. ^∗∗∗^*p* < 0.001, (a), (g), and (l) scale bar = 20 *μ*m; (c), (j) scale bar = 50 *μ*m, *n* = 3.

Previous studies have shown that CD137 is a key regulator in the progression of atherosclerotic plaque [36, 37]. Activation of CD137 signaling in ECs or macrophages has been shown to contribute to intraplaque angiogenesis [12, 13]. To further investigate the effects of CD137 on the angiogenesis process, we evaluated CD31-positive microvessels in CD137^−/−^ ApoE^−/−^ mice and sprouting in the aortic rings in CD137^−/−^ mice. The number of microvessels expressing CD31 was lower in the CD137^−/−^ group compared to the control mice (Figures 1(a) and 1(b)). As shown in Figures 1(c) and 1(d), fewer sprouts were observed in the CD137^−/−^ApoE^−/−^ group. In order to verify the important role of CD137 molecule, ECs were transfected with CD137 siRNA, and the silencing efficiency was detected by western blot (Figures 1(e) and 1(f)). After treatment with human siCD137, the total length of HUVEC tubes and number of sprouting branch points decreased significantly compared with the control group. Furthermore, we transfected mouse brain microvascular ECs (BMVECs) with murine siCD137 and observed a lower ratio of ECs proliferation (Figures 1(k) and 1(j)) and a reduced number of migratory cells.

### 3.2. Ligation of CD137 with CD137L Influences the VEGFR2 Expression and Activates Downstream Akt/eNOS

**Figure 2:**
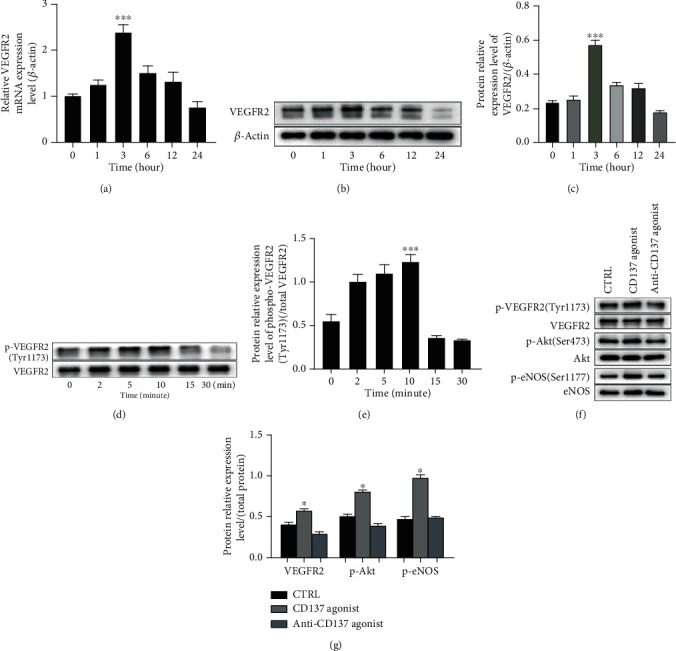
Activation of CD137 with CD137L influences the expression of VEGFR2 and activates downstream Akt/eNOS. (a) The mRNA level of VEGFR2 by RT-PCR peaked at 3 hours after treatment with CD137L (10 *μ*g/ml) at 0, 1, 3, 6, 12, and 24 hours, respectively. (b) The VEGFR2 expression measured by western blot, and protein relative level standardized by *β*-actin (c). (d) Phosphorylated VEGFR2 (p*-*VEGFR2, Tyr1173, murine) at 0, 2, 5, 10, 15, and 30 minutes, and the relative protein expression normalized by total VEGFR2 (e). (f) p-VEGFR2, p-AKT, and p-eNOS were detected by western blot under activating and blocking the CD137 signal for 10 minutes and standardized by total protein, ^∗^*p* < 0.05, the CD137 agonist group compared with the control or anti-CD137 group (g). ^∗∗∗^*p* < 0.001, *n* = 3.

Although activation of CD137 signaling contributed to angiogenesis, the potential key molecules involved in this process are still unclear. In this context, we screened a series of angiogenesis-related candidates and revealed that activation of CD137 signaling with recombinant human CD137L affected the mRNA levels of several molecules, including VEGFR2 (Figure 2(a)), Neuropilin-1, EphrinB2, DLL4, and Notch-1 (Supplementary Figure 1). Among these, VEGFR2 showed the highest expression (both transcription and translation levels) 3 hours after treatment with CD137L (Figures 2(a)–(c)). VEGFR2 is essential for both proliferation and differentiation of ECs [38]. Whether the VEGFR2 signal is activated, we detected the phosphorylation level of VEGFR2 after activating CD137 signaling for a time point, 0, 2, 5, 10, 15, and 30 minutes and found that phosphorylated VEGFR2 (Tyr1173) was increased, achieving the maximum level at 10 minutes, which indicated that CD137 rapidly promoted VEGFR2 phosphorylation at Tyr1173 (Figures 2(d) and 2(e)). Besides, activating the CD137 signal for 10 minutes promoted Akt and eNOS phosphorylation (Figures 2(f) and 2(g)) both the downstream molecules of phosphorylated VEGFR2 [39]. And blocking CD137 signaling with inhibitory anti-CD137 antibody weakened the effects of CD137 signaling on VEGFR2 and Phospho-Akt (p-Akt, Ser473) and phospho-eNOS (p-eNOS, Ser1177) (Figures 2(f) and 2(g)). These data suggest that the CD137 signal may be an additional way to affect the VEGFR2 expression and activation.

### 3.3. Inactivation of VEGFR2 Impairs CD137-Induced EC Proliferation and Migration

**Figure 3:**
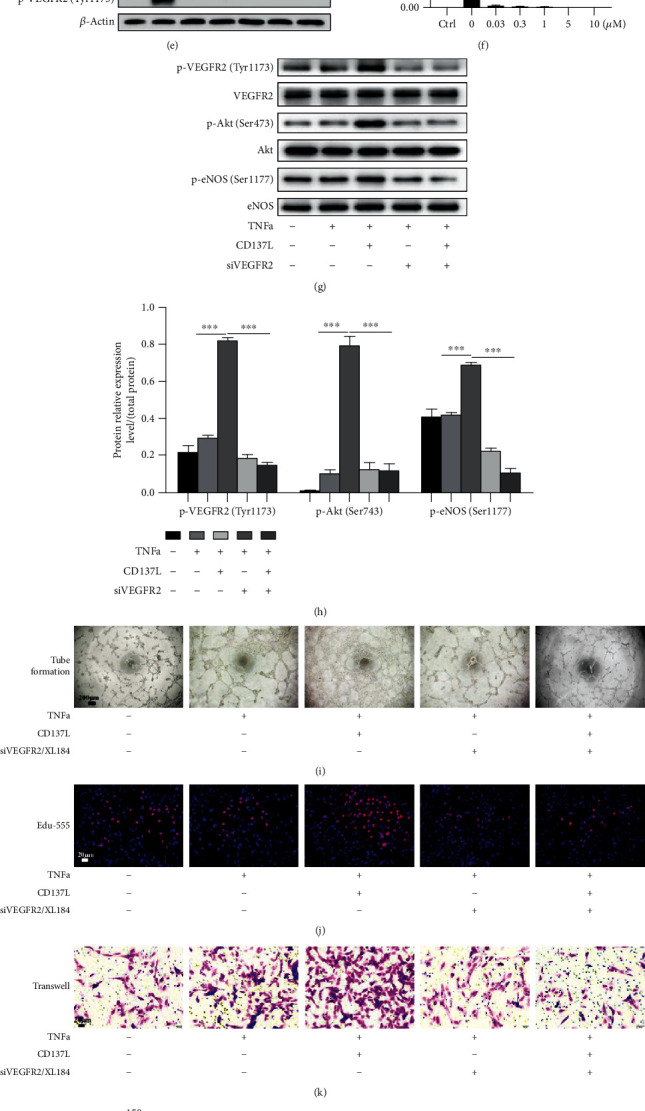
Silencing VEGFR2 impaired CD137 signaling-induced the Akt/eNOS pathway activation, endothelial cell proliferation, migration, and tube formation. (a, b) The protein expression of p-VEGFR2(Tyr1173) after treatment with VEGF165 (20 ng/mL) at 5, 10, 15, and 20 minutes. (c) The silencing efficiency of CD137 VEGFR2-1,-2,-3 (50 nM) after treatment with VEGF165 (20 ng/mL) detected by western blot. (e) After stimulation with VEGF165 (20 ng/mL) for 5 minutes, the effects of different concentrations of XL184 (cabozantinib, an inhibitor of VEGFR2 which was used in tube formation assay) at 0, 0.03, 0.3, 1, 5, and 10 *μ*M on p-VEGFR2(Tyr1173) were examined. (b, d, f) The protein relative expression level to *β*-actin. (g) Detection of p-VEGFR2(Tyr1173), p-Akt(Ser473), and p-eNOS(Ser1177) proteins after activating CD137 signaling by western blot after the CD137 activation for 10 minutes or/and inhibition of VEGFR2 with siVEGFR2. (h) The protein relative expression level of p-VEGFR2 (Tyr1173), p-Akt (Ser473), and p-eNOS (Ser1177) to total protein. (i) The tube formation of HUVECs after activation of CD137 alone or combined with inhibition of VEGFR2. The number of branch points and total length of tubes were observed under microscope and quantified by Image-Pro Plus 8.0; scale bar = 200 *μ*m (l, m). (j) The EdU-555 proliferation assay was used to detect endothelial proliferative ability after activation of CD137 signaling for 3 hours(n); red and blue fluorescence represent the proliferating cells and nuclei, respectively. (k) Endothelial cells were cultured in 24-well transwell plates (pore size: 8 *μ*m) for 12 hours after treatments, and transwell assay was applied to detect endothelial migratory ability (o); the violet color represents migrating cells stained with 0.1% crystal violet. The numbers of proliferative and migratory ECs were analyzed with Image-Pro Plus 8.0 (n, o). ^∗∗∗^*p* < 0.001, scale bar = 20 *μ*m, *n* = 3.

EC migration and proliferation contribute to angiogenic sprouting [32]. Since VEGFR2 plays an important role in angiogenesis [31, 40, 41], it is necessary to investigate whether the CD137-dependent enhancement of the EC angiogenic function occurred through VEGFR2 signaling. In order to observe the silencing efficiency of siVEGFR2, VEGFA165 (20 ng/mL) [42], the most studied VEGFA isoform, was used to upregulate the VEGFR2 expression at different time points. The protein level of p-VEGFR2 (Tyr1173) obviously increased after stimulation with VEGFA for 5 minutes (Figures 3(a) and 3(b)). Subsequently, siVEGFR2 (50 nM) was transfected into ECs, and the knockdown efficiency of siVEGFR2-2 was verified by western blot (Figures 3(c) and 3(d). Meanwhile, XL184 (a potent inhibitor of VEGFR2, 0.03 *μ*M) was incubated with HUVECs and aortic rings (Figures 3(e) and 3(f)). In accordance with previous studies [8, 12], we preincubated ECs with TNF*α* (10 ng/mL) to increase the CD137 expression on the cell surface before treatment with CD137L. As shown by western blot, activation of CD137 signaling with CD137L significantly increased phosphorylation of VEGFR2(Tyr1173), p-Akt(Ser473), and p-eNOS(Ser1177), whereas silencing of VEGFR2 abolished the effect of CD137 activation (Figures 3(g) and 3(h).

Next, we performed the EC tube formation assay to observe the endothelial function. Compared to TNF*α* treatment alone, capillary-like structures with a higher number of branches and greater total length of tube network were observed after incubation with additional CD137L for 3 hours. Silencing of VEGFR2 markedly weakened the angiogenic ability of ECs induced by the CD137 signaling activation with less connected ECs and less well-formed tubes. In contrast, incubation with CD137L did not abrogate the effect of VEGFR2 inhibitors (Figures 3(i), 3(l), and 3(m)). In addition, the results of endothelial Edu-555 and transwell assays showed that VEGFR2 knockdown notably decreased CD137-induced endothelial proliferation and migration. Consistent with the results on tube formation, added CD137L did not reverse the EC function induced by VEGFR2 inhibitors (Figures 3(j), 3(k), 3(n), and 3(o)).

### 3.4. CD137 Signaling Promotes the Endothelial Function via the VEGFR2/Akt/eNOS Pathway

Phosphorylated VEGFR2 at 1173 tyrosine residue transduces the signaling of cell proliferation and migration [31]. Besides, studies have reported that the VEGFR2-dependent activation of PI3K-Akt signaling regulates cell survival [26, 43]. Akt is a key molecule in that signaling pathway, which is critical for VEGFR2 functioning. Moreover, eNOS activated by Akt regulates cell permeability.

**Figure 4:**
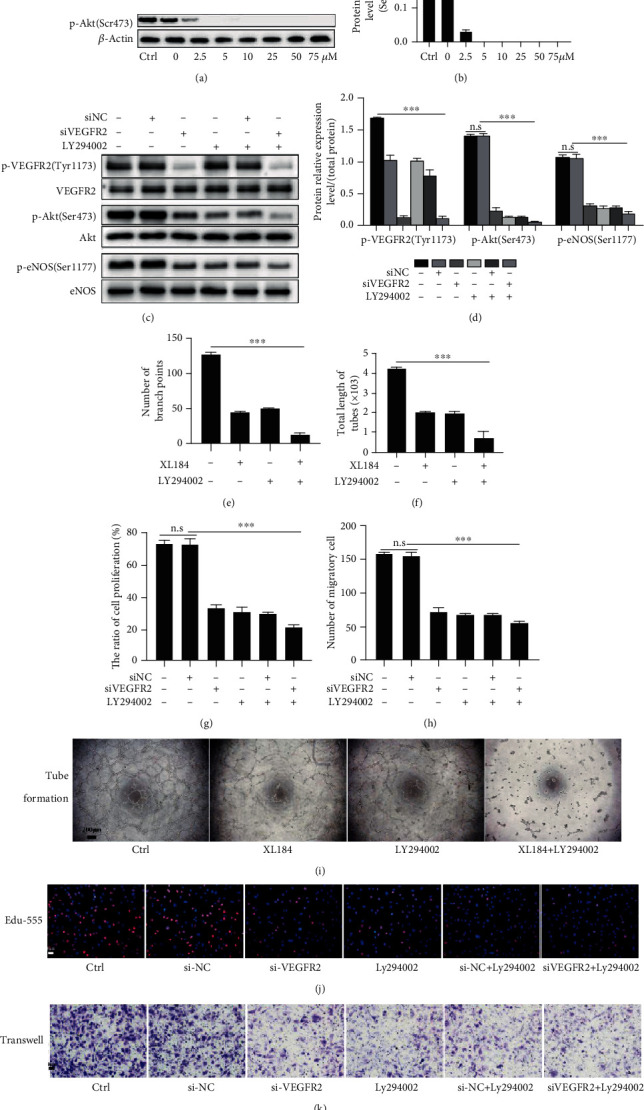
Inhibition of the VEGFR2/Akt/eNOS pathway attenuated CD137 signaling activation-mediated endothelial function. (a, b) The relative protein expression of p-Akt(Ser473) after treatment with different concentrations of LY294002 (0, 2.5, 5, 10, 25, 50, and 75 *μ*M) for 30 minutes and analyzed to *β*-actin. c Detection of p-VEGFR2 (Tyr1173), p-Akt (Ser473), and p-eNOS (Ser1177) proteins by western blot after treatment with VEGFR2 siRNA or/and LY294002 then activation of CD137 signaling for 10 minutes. The protein relative expression was quantified to total proteins (d). (i) The tube formation of HUVECs after treatment with VEGFR2 siRNA or/and LY294002 with the CD137 signaling activation. Scale bar = 200 *μ*m (e, f). (j, k) the EdU-555 proliferation assay and transwell assay were applied to detect endothelial proliferative and migratory ability. (j, k) Red and blue fluorescence represent the proliferating cells and nuclei, respectively, and the violet color represents migrating cells stained with 0.1% crystal violet. (g, h) The numbers of proliferative and migratory ECs were analyzed with Image-Pro Plus 8.0. ^∗∗∗^*p* < 0.001. All treatments were under the circumstance of the CD137 signaling activation. Scale bar = 20 *μ*m, *n* = 3.

From the above results, CD137 signaling activated p-VEGFR2 and p-Akt and increased the p-eNOS protein expression. Therefore, we investigated whether the VEGFR2/Akt/eNOS pathway was indispensable for the CD137 signaling-mediated endothelial tube formation, proliferation, and migration. We applied LY294002 (an inhibitor of p-Akt) to verify the downstream signaling transduced by CD137-CD137L. First, we found that LY294002 (5 *μ*M) obviously inhibited the p-Akt (Ser473) expression (Figures 4(a) and 4(b)). Compared with the group without inhibitors, siVEGFR2 impaired the expression of phosphorylated VEGFR2 (Tyr1173), p-Akt (Ser473), and p-eNOS (Ser1177). Treatment of LY294002 decreased the expression of p-Akt (Ser473), while it did not affect the level of p-VEGFR2. With simultaneous addition of siVEGFR2 and LY294002, there was a marked reduction in p-VEGFR2 (Tyr1173) or p-Akt (Ser473) in comparison with siVEGFR2 or LY294002 alone (Figures 4(c) and 4(d)).

The tube formation was significantly impaired after treatment with siVEGFR2 or LY294002, showing less branches points and a reduced total length of tubes. Combination of VEGFR2 silencing and LY294002 could achieve a synergistic effect on angiogenesis (Figures 4(i), 4(e), and 4(f)). Similarly, either siVEGFR2 or LY294002 decreased EC proliferation and migration induced by CD137 signaling. Correspondingly, siVEGFR2 combined with LY294002 synergistically inhibited proliferation and migration (Figures 4(j), 4(k), 4(g), and 4(h)).

### 3.5. CD137 Signaling Enhances Aortic Sprouting through the VEGFR2/Akt/eNOS Pathway

**Figure 5:**
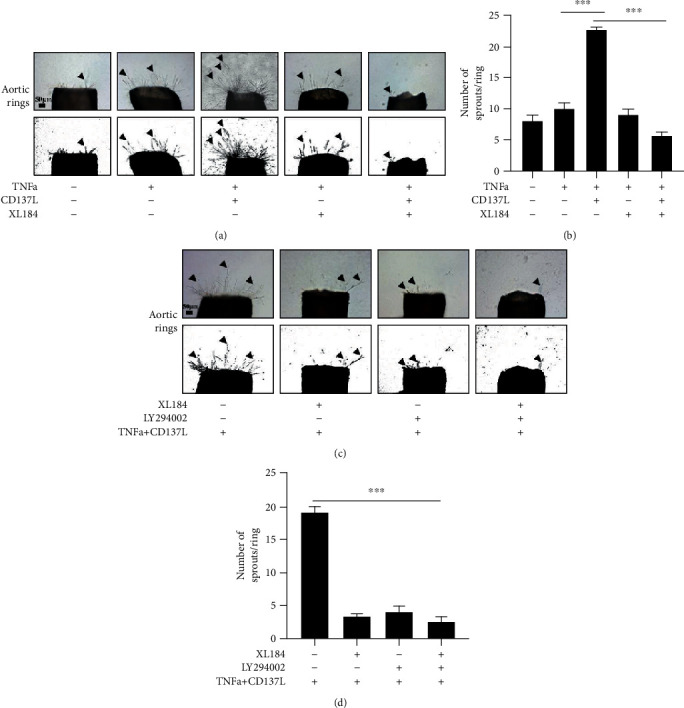
CD137-enhanced aortic sprouting and outspreading through the VEGFR2/Akt/eNOS pathway. (a) The sprouting of aortic rings under the CD137 activation or/and inhibition of VEGFR2 with XL184 (cabozantinib). (b) Number of sprouts per ring was quantified by Image-Pro Plus 8.0, scale bar = 50 *μ*m. (c) Aortic rings sprouting after treatment with VEGFR2 siRNA or/and LY294002 under the CD137 activation. (d) Number of sprouts per ring was quantified by Image-Pro Plus 8.0, scale bar = 50 *μ*m. ^∗∗∗^*p* < 0.001, *n* = 5.

VEGFR2 is highly expressed on tip cell filopodia which is responsible for guiding and migrating to an avascular region during neovessel formation [26, 27, 31, 44, 45]. Since VEGFR2 controls the migration of tip cells, it is important to figure out whether CD137 signaling induces VEGFR2 and downstream pathway implicated in aortic ring sprouts. We performed the mouse aortic ring assay, which revealed that CD137 signaling promoted budding of aortic rings as evidenced by a higher number of sprouts. Inhibition of VEGFR2 with XL184 impaired the proangiogenic sprouting mediated by CD137 signaling (Figures 5(a) and 5(b)). Additionally, inhibition of VEGFR2 and/or Akt decreased the number of sprouts, suggesting weakened sprouting capability of aortic rings (Figures 5(a) and 5(b)). Moreover, the capability of the aortic ring sprouting was attenuated considerably in the XL184 plus LY294002 group (Figures 5(c) and 5(d)), suggesting that sprouts were significantly reduced after pretreatment with XL184, LY294002, or both, before activation of CD137 signaling.

### 3.6. Inhibition of eNOS Weakens Angiogenesis Mediated by CD137 Signaling

**Figure 6:**
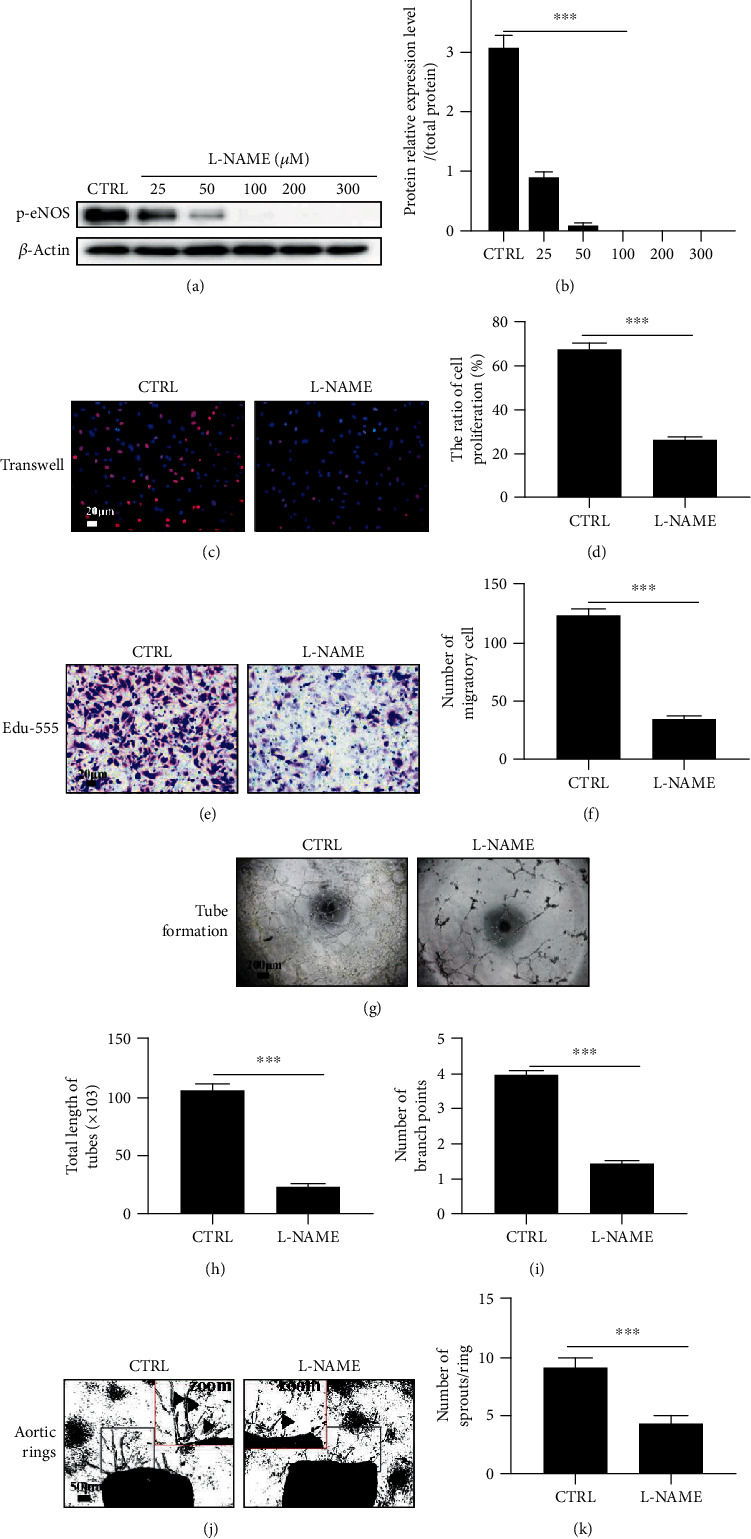
Inhibition of the eNOS activity weakened CD137 signaling-mediated angiogenesis in vitro and ex vivo. (a) The p-eNOS protein level was detected by western blot after treatment with different concentrations of L-NAME (0, 25, 50, 100, 200, and 300 *μ*M) for 24 hours. (b) The p-eNOS protein relative level was standardized by *β*-actin. (c, e) Treatment with L-NAME before activating CD137 signaling for 3 hours, then the EdU-555 proliferation assay and transwell assay were performed. (d, f) Proliferation and migration cells were detected and counted by red nuclei and the violet cell, respectively. (g) The tube formation of HUVECs was observed after treatment with L-NAME(100 *μ*M) for 30 min under the CD137 signaling activation. (h, i) Total length of tubes and number of branch points, scale bar = 200 *μ*m. (j) Aortic rings sprouting after pretreatment with L-NAME(100 *μ*M) for 24 hours before activation of CD137. (k) Number of sprouts per ring(black arrows) was quantified by Image-Pro Plus 8.0, scale bar = 50 *μ*m, *n* = 3.

To explore the potential effects of eNOS activation on angiogenesis mediated by CD137 signaling, we applied L-NAME (eNOS inhibitor) to inhibit the eNOS activity (Figure 6(a)). Specifically, we pretreated the ECs and aortic rings with L-NAME (100 *μ*M) before activating CD137 signaling. In vitro, both Edu-555 proliferation and transwell assay showed that L-NAME decreased the proliferative and migratroy cells compared with the control group (Figures 6(d) and 6(f)). Besides, the endothelial tube formation assay displayed the same tendency, showing a decreased total length and branch points of tubes in the L-NAME-treated group (Figures 6(h) and 6(i)). Moreover, the aortic ring assay indicated fewer sprouts compared with controls (Figure 6(k)).

### 3.7. Activation of CD137 Signaling Increases the EC Secretion of VEGFA

VEGFR2 is a member of VEGF receptors (VEGFR-1, -2, -3), highly expressed in migratory ECs (tip cells) filopodia, and senses VEGF [46]. VEGFA/VEGFR-2 is likely the prominent signaling that mediates cellular responses involved in angiogenesis [31]. VEGF binding to VEGFR2 initiates VEGFR2 dimerization and autophosphorylation at tyrosine kinase residues [47, 48], which is essential for downstream signaling transduction. To figure out whether CD137-mediated increase in VEGFR2 was related to VEGF, we incubated CD137-activated ECs either with VEGFA or with supernatant from ECs treated with CD137L, for 3 hours (Figure 6(a)). Since VEGF is a secreted protein, we speculated that CD137-dependent secretion of VEGFA would be verified by the similar effect of VEGFA on ECs (Figures 6(b) and 6(c)). Furthermore, the VEGFA expression of EC extracts was obviously decreased in the CD137L group (Figures 6(d) and 6(e)), while neutralized the CD137 signaling with inhibitory antibody increased the VEGFA level. The concentration of VEGF in concentrated condition medium of the CD137L group was significantly increased (Figures 6(f)–(h)), while anti-CD137 signaling decreased the VEGFA concentration. Meanwhile, the VEGFA expression in exosomes (extracted as the representatives from the condition medium as previously described [24]) was obviously lower in the CD137L group in comparison with the controls (Figures 6(i) and 6(j)). Inhibition of the CD137 signaling reversed this decrease. These results indicated that activation of CD137 signaling with CD137L promoted endothelial VEGFA secretion.

Taken together, this study indicated that CD137 signaling promoted sprouting angiogenesis through activation of the VEGFR2/Akt/eNOS pathway.

## 4. Discussion

Targeting intraplaque angiogenesis may be an important strategy in slowing down the progression of atherosclerotic diseases. Here, we investigated mechanisms of CD137-mediated angiogenesis. Specifically, this study showed that activation of CD137 signaling promoted sprouting angiogenesis in vitro and ex vivo, suggesting that blockage of CD137 may reduce angiogenesis through decreased VEGFA/VEGFR2 signaling. Therefore, combination of CD137 inhibitor and antiangiogenic agents could be an attractive strategy for treating atherosclerosis.

Sprouting angiogenesis is crucial both in physiological and in pathological circumstances, such as ischemia, inflammation, diabetes, and cancer [49]. Interaction of various factors and cells takes place in pathological angiogenesis that occurs in pathogenesis of atherosclerosis. Apart from providing nutrients and oxygen needed for the thickened arterial intima caused by excessive cellular proliferation, angiogenesis also provides a pathway for inflammatory mediators to enter the plaque, thereby reducing the plaque's stability, and increasing the risk of rupture and consequent adverse cardiovascular events [50, 51].

As an immunostimulatory receptor and inflammation regulator, CD137 plays a critical role in pathogenesis of atherosclerosis [10]. CD137 is activated and highly expressed on ECs under inflammatory conditions of atherosclerosis [7]. Its ligand (CD137L), which is expressed by activated antigen-presenting cells (APCs), binds to CD137 on ECs and promotes angiogenesis. We previously demonstrated that the CD137 signaling activation promoted angiogenesis in plaque [12, 13]. CD137 knock-out mice showed the essential effect of CD137 on sprouting angiogenesis as evidenced by a significantly decreased number of microvessels in the plaque and sprouts in aortic rings. Assays on ECs in vitro also suggested the importance of CD137 for endothelial proliferation and migration, as well as for the tube formation (Figure 1). Together, these data showed that CD137 was critical for angiogenesis.

Next, we intended to explore the mechanisms of CD137-mediated angiogenesis in vascular inflammatory milieu. Olofsson [7] reported that TNF*α* alone induced the strongest mRNA expression of endothelial CD137, which was also verified in our previous study [12]. In this context, we used TNF*α* as the basic treatment for ECs to improve the CD137 protein expression before stimulation with CD137L. Although the relationship between CD137 signaling and angiogenesis has been poorly studied, activation of CD137 signaling could promote angiogenesis in atherosclerosis through modulating the endothelial smad1/5-NFATc1 pathway [12]. Moreover, in an indirect way, CD137 signaling mediates phenotypic conversion of macrophages to influence angiogenesis [13]. It remains unknown whether there is an obvious factor involved in CD137 signaling-mediated angiogenesis. We screened lots of angiogenesis-related candidates at the transcriptional level and found that VEGFR2, the critical regulator of angiogenesis, was obviously increased both at transcriptional and translational levels. In addition, considering the complex role of VEGFR2 in atherosclerosis, regulation of VEGFR2 through CD137 signaling might be crucial for the neovessel formation. The high expression of VEGFR2 on EC surfaces provided broader access for VEGF binding [52], although some studies reported that VEGFR2, if retained on cell membrane for a long time, inhibited VEGF's binding and inactivated the downstream transduction. The VEGFR2 protein expression measured in ECs which treated with CD137L for a shorter time course, as indicated in Figure S2 (Supplementary materials), suggested a substantial upregulation of VEGFR2 which may contribute to activation VEGFR2 signaling [51]. CD137 activated the phosphorylated VEGFR2 at Tyr1173 (Figure 2). Additionally, p-Akt and p-eNOS changed synchronously with p-VEGER2. Moreover, our group has demonstrated that the CD137 signaling activated Akt in ECs in atherosclerosis [53].

Moreover, endothelial NO synthase (eNOS) is produced by ECs and reflects endothelial activities. A low level of eNOS is considered essential for maintaining the endothelial functions [54]. The deficiency in eNOS markedly decreased retinal neovascularization in a mouse model [55]. Additionally, Huang et al. reported that soluble delta-like 1 homolog (DLK1) stimulated angiogenesis through Notch1/Akt/eNOS signaling in ECs [39]. CD137 signaling activating eNOS conforms to the studies on CD137 proangiogenesis effects [12, 13].

To further investigate whether the effects of CD137 signaling on EC functions are mediated by VEGFR2, we transfected ECs with siVEGFR2 or XL184 to block VEGFR2. VEGFA165 was used as an inducer of VEGFR2 before siVEGFR2 transfection. Inhibition of VEGFR2 weakened the Akt and eNOS activation expression and reduced endothelial proliferation and migration mediated by CD137 signaling (Figure 3).

Decreased p-eNOS and unchanged VEGFR2 protein level after inactivating Akt during the CD137 signaling activation suggest that Akt and eNOS are the downstream molecules of VEGFR2 signaling (Figure 4). Likewise, the proliferative and migratory functions were dampened when inhibiting Akt under the CD137 signaling activation condition [56]. These results are consistent with the previous studies [26, 31, 57, 58].

VEGFR2 is critical for migratory ECs that sense proangiogenic factors. Both Akt and eNOS are essential for survival and migration of ECs. In line with in vitro results, the aortic ring assay showed that the number of sprouts per ring increased obviously when treated with CD137L. The prosprouting effect was significantly impaired after inhibition of VEGFR2 or/and Akt (Figure 5). Besides, inhibition the activity of eNOS weakened the effects of CD137 on angiogenesis, suggesting that CD137-mediated angiogenesis is not only eNOS-related (Figure 6). Together, these results demonstrated the key role of the VEGFR2/Akt/eNOS pathway in CD137 signaling-mediated sprouting angiogenesis.

**Figure 7:**
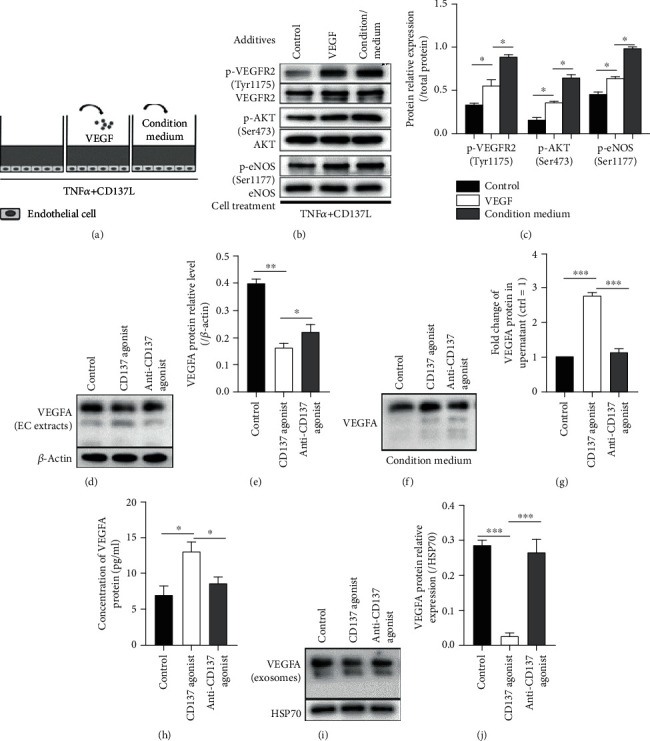
CD137 signaling increased endothelial secretion of VEGFA. (a) Schematic diagram of different treatments used on CD137-activated ECs. (b) Detection of endothelial p-VEGFR2 (Tyr1173), p-Akt (Ser473), and p-eNOS (Ser1177) proteins by western blot after coculture with VEGFA or condition medium for 3 h. (c) The relative expression level of p-VEGFR2 (Tyr1173), p-Akt (Ser473), and p-eNOS proteins. (d, e) Detection of the VEGFA expression from endothelial extracts in control and CD137L groups. The relative VEGFA protein level was quantified in relation to *β*-actin. (f, g) The VEGFA expression and relative change of concentrated condition medium in the control and CD137L groups. (h) VEGFA concentration of the condition medium was measured with ELISA detection after treatment with CD137L for 3 h. (i, j) The VEGFA expression in exosomes, which derived from the supernatant of the control and CD137L groups. ^∗^*p* < 0.05, ^∗∗^*p* < 0.01, ^∗∗∗^*p* < 0.001, n = 3. The CD137 agonist group was stimulated with TNF*α* for 24 hours and then CD137L for 3 hours. The Anti-CD137 group was treated with inhibitory CD137 signaling antibody for 30 minutes then CD137L for 3 hours. Condition medium contained the supernatant after the activation of endothelial CD137 signaling for 3 hours.

Signal transduction networks initiated by VEGFA/VEGFR2 lead to EC proliferation, migration, survival, and new vessel formation. Cocultured CD137 signaling-activated ECs with VEGFA165 or the condition medium, which was from VEGFR2 highly expressing ECs, showed the activation of the VEGFR2/Akt/eNOS pathway. Preactivating CD137 signaling of cultured ECs could explain the expression of p-VEGFR2 and p-Akt in the control group. We subsequently analyzed whether CD137 signaling influenced the expression of VEGFA and showed that VEGFA in EC extracts was lower after activating CD137 signaling. However, the VEGFA protein level was higher in the condition medium as detected by western blot and ELISA, implying that VEGFA was secreted to the extracellular space. To verify whether VEGFA was carried to the extracellular space via vesicles, we examined VEGFA protein in endothelial exosomes and found that iy\t was significantly diminished after activating CD137 signaling (Figure 7). Furthermore, blocking the CD137 signaling reversed its effects on the VEGFA expression and secretion. Furthermore, previous studies demonstrated that PlGF potentiated the effects of VEGFA binding with VEGFR2 by stimulating VEGFA secretion [59, 60], thus exerting a proangiogenic and proatherogenic effect [61]. However, whether increased VEGFA secretion mediated by CD137 signaling that influences VEGFR2 signaling transduction needs more investigation.

In addition, the expression of CD137 was reported in a wide range of tumor cells [62] and tumor vessel walls [14]. While CD137 agonist immunotherapy is beneficial for cancer control and treatment [63, 64], the accompanying proangiogenic effects of CD137 activation may weaken anticancer therapy. Therefore, combining antiangiogenesis agents and immune checkpoint blockers would be an attractive strategy for cancer treatment [15], considering that the CD137 activation together with antiangiogenesis enhance the antitumor effects.

Here, we revealed that the CD137/CD137L costimulatory molecule activates VEGFR2 and influences downstream signaling transduction, which provides an avenue for combined immunological antiangiogenic therapy in angiogenic diseases. However, further studies are necessary to unravel the mechanisms of CD137-related VEGFA secretion and the effects of CD137 agonists combined with antiangiogenic agents on angiogenesis in vivo.

**Figure 8:**
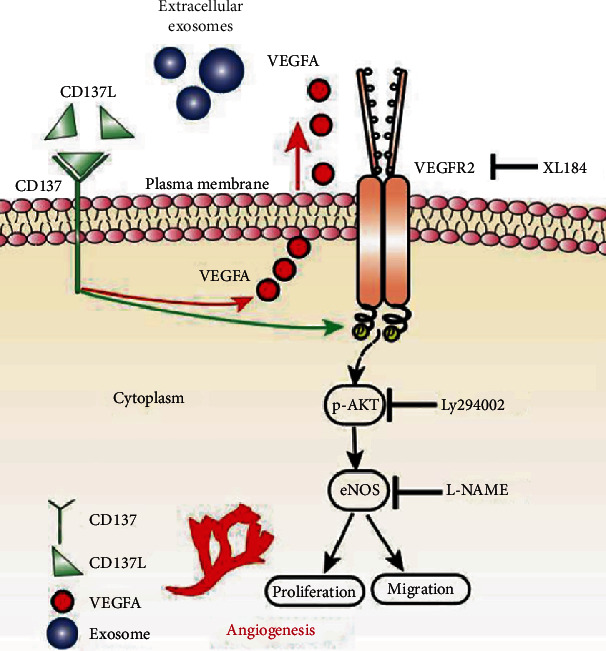
Graphical abstract. Activation of CD137 signaling promoted sprouting angiogenesis via increased VEGFA secretion and the VEGFR2/Akt/eNOS pathway. CD137 upregulated the expression of VEGFR2 on the filopodia of migrating endothelial cells to sense VEGFA in the milieu. Therefore, increased VEGFA secretion together with the VEGFR2 activation induced downstream Akt/eNOS transduction, leading to proliferation, migration, and angiogenesis.

In summary, the activation of CD137 signaling promotes sprouting angiogenesis by the increased VEGFA secretion and VEGFR2/Akt/eNOS pathway (Figure 8). CD137 signaling and VEGFA-VEGFR2 may be the potential targets for intraplaque angiogenesis. Moreover, combination of CD137 agonists with specific VEGFA/VEGFR2 inhibitors may be effective for angiogenesis in tumors.

## Data Availability

The data used to support the findings of this study are available from the corresponding author upon request.

## Supplementary Materials

Supplementary Materials Primers used in the RT-PCR assay: VEGFR2-forward-murine, gtc atg gat cca gat gaa ttg c; VEGFR2-reverse murine, tct tgt caa ttc caa aag cgt c; *β*-actin-forward murine, gtg cta tgt tgc tct aga ctt cg; *β*-actin-reverse murine, atg cca cag gat tcc ata cc.
