# Supplementary material for: Activating CD137 Signaling Promotes Sprouting Angiogenesis via Increased VEGFA Secretion and the VEGFR2/Akt/eNOS Pathway
Source: Mediators Inflamm. 2020 Oct 24;2020:1649453. doi: 10.1155/2020/1649453 (PMC7604604; doi:10.1155/2020/1649453)
Supplement: Supplementary Materials — Primers used in the RT-PCR assay: VEGFR2-forward-murine, gtc atg gat cca gat gaa ttg c; VEGFR2-reverse murine, tct tgt caa ttc caa aag cgt c; β-actin-forward murine, gtg cta tgt tgc tct aga ctt cg; β-actin-reverse murine, atg cca cag gat tcc ata cc. [file 1649453.f1.docx]

**
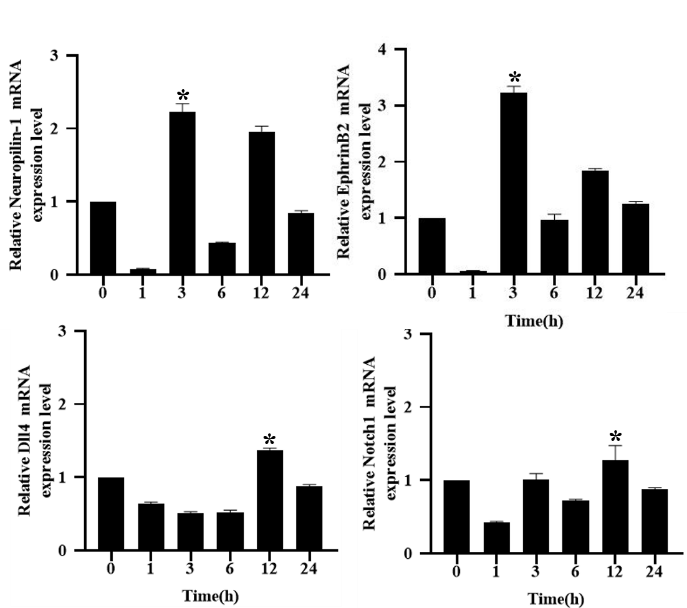
Supplementary Figure 1**

The relative mRNA level of four other angiogenic molecules(Neuropilin-1, EphrinB2, DLL4, and Notch-1) implicated in VEGFR2 signaling was detected by RT-PCR assay, *p<0.05.


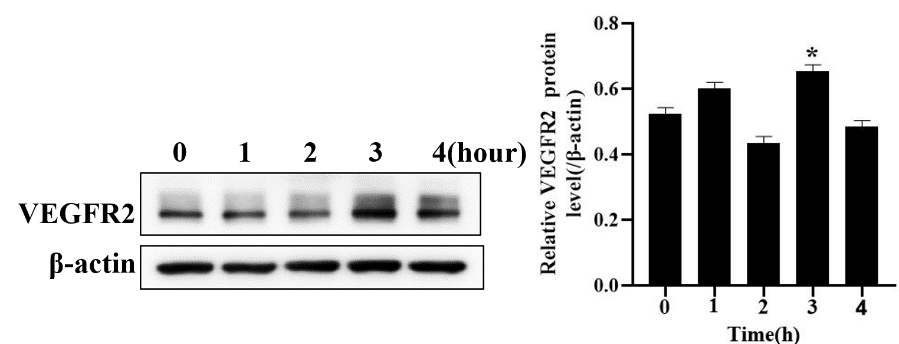
**Supplementary Figure 2**

Activation of CD137 signal with CD137L at different shorter time points at 0,1,2,3, 4hour respectively. VEGFR2 expression was peaked at 3hours which was consistent with the longer time points. VEGFR2 protein bands measured by western blot(right). Analyzed of relative VEGFR2 protein expression (left histogram, *p<0.05)


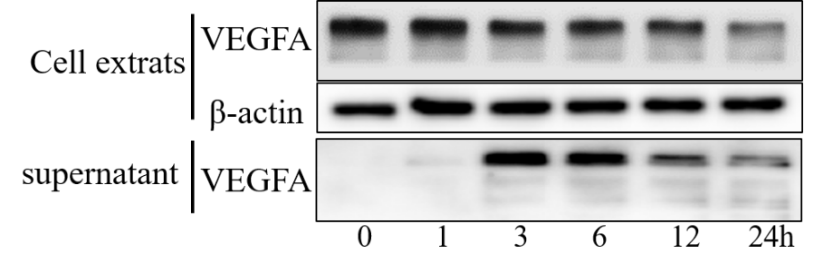
**Supplementary Figure 3**

Activation of CD137 signal with CD137L at different time points at 0,1,3,6,12,24hour respectively. VEGFA protein expression from supernatant was peaked at 3hours and decreased at 3 hours from endothelial cell extracts which were consistent with the results in Figure 7d, 7f.

**Supplementary material**

Primers used in RT-PCR assay: VEGFR2-forward-murine, gtc atg gat cca gat gaa ttg c; VEGFR2-reverse murine, tct tgt caa ttc caa aag cgt c; b-actin-forward-murine, gtg cta tgt tgc tct aga ctt cg; b-actin-reverse-murine, atg cca cag gat tcc ata cc;

**Exosomes extraxtion and detection**

Exosomed derived from endothelial cells were exactrated as our previous study^[1]^.

**References**

[1] Li B, Zang G, Zhong W, et al., "Activation of CD137 signaling promotes neointimal formation by attenuating TET2 and transferrring from endothelial cell-derived exosomes to vascular smooth muscle cells," *Biomedicine & pharmacotherapy = Biomedecine & pharmacotherapie*, vol. 121, no., pp. 109593, 2020.
